# An Integrative Approach to the Study of Cognitive Abilities in a Non-Human Primate Model in a Virology Laboratory Environment

**DOI:** 10.3390/brainsci14070635

**Published:** 2024-06-26

**Authors:** Anastasia Rogova, Anna Kalyanova, Yulia Rogova, Maria Fedina, Alexandra Siniugina, Aydar Ishmukhametov, Galina Karganova

**Affiliations:** 1 FSASI “Chumakov FSC R&D IBP RAS” (Institute of Poliomyelitis), Moscow 108819, Russia; rogova94@icloud.com (A.R.); annakalyanova@bk.ru (A.K.); rogova-ju@rambler.ru (Y.R.); mariafedinamf@gmail.com (M.F.); sinyugina@chumakovs.su (A.S.); ishmukhametov@chumakovs.su (A.I.); 2 Institute of Translational Medicine and Biotechnology, Sechenov First Moscow State Medical University, Moscow 119991, Russia

**Keywords:** behavior, cognitive tests, cognitive function, training techniques, positive reinforcement training, non-human primates, long-tailed macaques, virus infection

## Abstract

Non-human primates, due to their similarities in immune response to humans, are the preferred model for studying infectious processes and any associated cognitive impairments. Behavioral tests are indispensable for investigating pathogenesis in neuroinfections, especially those that do not manifest with noticeable clinical symptoms, as well as in the transition to a chronic form of the disease. Modeling viral infection requires specialized experimental conditions. Our work describes techniques for investigating mnemonic functions, tiredness, attentional focus, quick-wittedness, and basic behavioral responses in primates under the assumed conditions for infections with viruses that do not have an airborne route of transmission. It also outlines approaches to the training and selection of primates for virological research, as well as analyzing gender differences in learning abilities, the impact of housing conditions on the results, and the correlation between training success and behavioral test scores. These methods will allow a more detailed study of non-human primates as a model for researching cognitive and behavioral impairments under infectious and immune stress, as well as the design of less energy-intensive experiments for evaluating the efficacy and safety of therapeutic and prophylactic strategies at early stages of infection.

## 1. Introduction

Choosing an animal model is one of the critical points in the design of experimental studies aimed at investigating cognitive abilities and behavioral responses. Long-tailed macaques (*Macaca fascicularis*) are the predominant primate species for preclinical trials of biopharmaceutical safety and the modeling of pathological conditions, including known techniques for testing cognitive abilities in this primate species [1,2]. This primate model holds great prospects for researching common and debilitating diseases associated with neurotropic viruses and identifying new therapeutic targets when dealing with the consequences of viral neuroinfections.

Recently, there has been a growing interest among scientists in understanding how viral infections of the nervous system affect behavior and cognitive function [3,4,5,6,7,8]. For most viral infections, there is a significant predominance of asymptomatic infection over acute infection. Many infections may proceed with minimal or no symptoms at all, complicating timely diagnosis and potentially leading to long-term neuropsychological disorders. This may be due to the direct action of the viral agent, the body’s immune response to the pathogen, or the result of organ damage [9]. In this context, cognitive tests serve as an important tool for detecting subtle changes in cognitive status. As asymptomatic infections involve clinically non-evident disturbances, an informative and sensitive model is necessary. Primates are the most promising animal model closely resembling human immune responses and are often used both for the study of virological aspects and for the screening of cognitive impairments [10,11].

Neurocognitive tests are widely used around the world for assessing memory status and attentional focus, as well as depressive, psychological, and mental disorders [12,13]. Various animal models, such as mice, rats, higher and lower primates, etc., are also used for testing pharmaceuticals and therapeutic approaches, including the successful modeling of diverse central nervous system (CNS) pathological disorders [14,15,16]. Among these, research on the effects of viral infections on the CNS and behavior holds significant relevance. However, apparently, due to the complexity of implementing such tests on primates, there is a noticeable lack of literature on this topic.

Before proceeding directly to testing the models, it is essential to consider many specifics of such scientific research, such as the following: the preliminary training and preparation of primates for behavioral tests; the availability of the proposed tests; the adaptation of animals to stressful conditions arising from interaction with experimenters and the application of various medical manipulations; the effect of these factors on the results of the experiment, as well as the conditions of animal housing; sex differences; and the temperament peculiarities of each individual primate. This list should include qualitative characteristics for selection into test groups. Lengthy and energy-intensive experiments underline the importance of carefully selecting animals at the initial stages of research without any subsequent exclusions of specific individuals due to their inability to perform the necessary tasks and/or untrainability.

In the context of working with infectious material, primates can experience a multitude of potential stressors, including physical restraints, injections, and solitary housing during virological studies, as well as being housed with other animals in the same room with the possibility of observing other primates and the manipulations performed on them. Training in voluntary co-operation using positive reinforcement training (PRT) methods is one way to significantly reduce the adverse effects of such procedures and housing factors on primates and is often used by researchers as a valid and humane approach [17,18]. For a comprehensive study of the impact of neurotropic viruses on the behavior and cognitive abilities of non-human primates, it is necessary to select and optimize training techniques and perform group selection and test selection specifically adapted for these conditions.

In order to achieve reliable results and a comprehensive understanding of modeled infectious diseases, including those caused by viruses, it is critically important not only to test on a wide sample of animals but also to ensure the strict synchronization of experiments conducted at the same stages for all animals. This is because the picture of the infection at different stages often differs significantly, and the animals are housed in the same facility. In planning the program for testing the cognitive abilities of primates, we relied on already known and proven methodologies, such as the Primate Cognition Test Battery (PCTB) [19], as well as on previously described approaches for studying performance disruption, by which tiredness can be assessed [20], and methods to study primates’ natural reaction to novelty [21] and their ability to solve complex intellectual tasks [22]. One of our main objectives was to select and modernize existing tests to make the training program and the experimental process easier for both researchers and the primates themselves. Additionally, we aimed to assess the initial results of the study of the primates before and after the influence of various factors, whereas most programs are designed for a single time point.

Previously, we conducted experiments to assess the impact of asymptomatic orthoflavivirus infection on the behavior and cognitive abilities of *Macaca fascicularis* [23]. Concurrently with the training of the primates, we conducted experiments to evaluate their cognitive capabilities, which were based on various cognitive functions, such as memory, attentional focus, spatial perception, and decision-making, and we encountered the situation that only a portion of the primates from the study group were ready to co-operate and were capable of performing intellectual tasks. During the training process, we faced the fact that a significant portion of the animals were unable to perform tasks and interact with experimenters; they also did not succeed in passing cognitive tests or outright refused any interaction with the experimenter. As a result, we realized the scale of cognitive ability variances among the primates and the need for a preliminary selection from the group.

In this work, we present techniques that will allow for the effective training of non-human primates and prepare them for conditions involving virological studies, and we propose markers for the exclusion of “non-functional” individuals at the early stages of training. 

## 2. Materials and Methods

### 2.1. Animals and Special Conditions

Sexually mature female (*n* = 9) and male (*n* = 10) long-tailed macaques (*Macaca fascicularis*) were used as the study model. The animals were received from the Klenovo Veterinary Station (Klenovo Settlement, Klenovo Village, Centralnaya Street), with the veterinary certificate issued by SBBZh TINAO (Animal Disease Control Station), Moscow, Novofedorovskoye Settlement. The 2–3-year-old macaques weighed between 1.6 and 3.5 kg. Test animals were kept in quarantine for 1 month before the experiment and posted in a specialized vivarium of the FSASI “Chumakov FSC R&D IBP RAS” (Institute of Poliomyelitis), in accordance with CIOMS recommendations, 1985, EU Directive 2010/63/EU and Appendix A to the European Convention ETS No. 123. The study protocol was approved by the Ethics Committee of the FSASI “Chumakov FSC R&D IBP RAS” (Institute of Poliomyelitis), protocol No. 211019-2, dated 21 October 2019. The animals’ diet was balanced with unlimited drinking water (ad libitum). The cycle of the day was 12 h of light and 12 h of darkness.

Virological studies on animal models, particularly on primates, require a sufficiently large number of animals in the test groups, as well as their individual housing. These facts indicate the necessity to create certain conditions for housing during the testing period. In this regard, we selected individual cages that were installed on a multi-tiered rack; such an approach allowed us to increase the number of animals within a single testing room space. All the animals were kept in individual cages located in the same room. Each 100 × 70 × 80 cm cage was equipped with a special sliding frame for fixing the animals. The rack design for installing individual cages allowed for three cage position options from the floor level: at human knee level, waist level, and head level. Based on point 3 of Article 33 of Directive 2010/63/EU of the European Parliament and of the Council on the protection of animals used for scientific purposes, changes in housing conditions for scientific purposes are permitted. Our experiment involved simulating the infection of macaques with viruses without an aerogenic transmission route, requiring BSL-3 conditions, and such preparation was critical for ensuring the safety of the experimenter during the manipulations with the animals (considering that the study of cognitive abilities excluded the use of anesthesia). The use of special mechanisms for holding the animals prevented the possibility of accidental bites or escape, which ensured the safety of both the experimenters and the primates and minimized their stress. The cages were equipped with a special platform for cognitive tests. In addition to this, we aimed to compensate for potential deficiencies and carefully enriched the animals’ living environment with cognitive (offering tasks and puzzles), dietary, and behavioral enrichment. The animals were kept in separate cages. The monkeys weighed 1.6–3.5 kg, and since the experiment was short-term, the smaller cage sizes did not negatively impact their well-being. This approach was approved by the Ethics Committee of the Scientific Center (protocol No. 211019-2 from 21 October 2019).

Qualified trained personnel implemented all manipulations according to the study protocol. The processes of conducting a behavioral test and training animals were carried out by two specialists who are competent in working with primates. The experimenters who conducted the behavioral tests did not perform any medical procedures on the primates to avoid causing negative impressions and reactions on the part of the animals. All medical procedures were carried out by other specialists with advanced veterinary degrees.

### 2.2. Experimental Design

**Figure 1:**
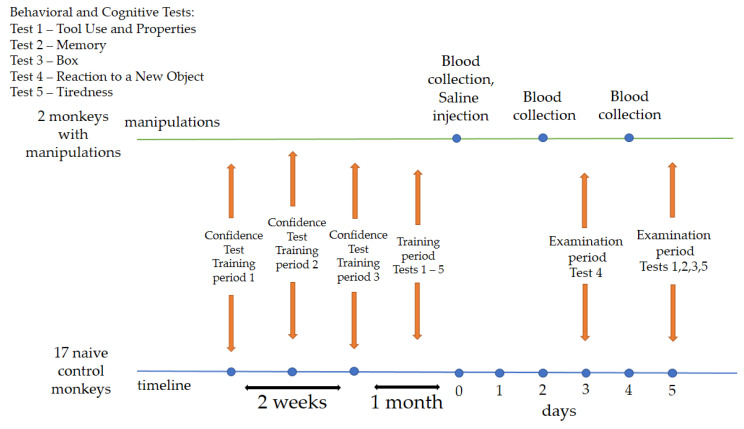
Overall experimental design. Chronology of the experimental process and procedures.

The training period was 1 month and 2 weeks. During the first two weeks, primates were asked to take the “Confidence” test. The program needed to be performed daily or with short breaks. We divided the “Confidence” test into 3 periods for summarizing, scoring, and assessing primate performance. Period 1: the first day of testing; period 2: results at the end of the first week of training; period 3: the last day of the “Confidence” test training. At the end of the “Confidence” test, the primates were administered cognitive–behavioral tests for one month (Tests 1–5). The program needed to be performed daily or with short breaks. After the training period, on day zero (0), two animals (saline) were administered a saline solution, followed by blood collection on days 2 and 4. Saline animals were selected through random number generation. On day 3 (examination period), all primates were asked to take Test 4. On day 5 (examination period), all primates were asked to take Tests 1, 2, 3, 5. Blood collection from the primates was carried out with a volume of 1.5–2 mL from the femoral vein using a 2.5 mL syringe with 23 G needles and without the use of preliminary anesthesia. A physiological solution (saline) of 0.25 mL was administered subcutaneously into the outer thigh area. The temperature in the groin area was measured daily in the primates receiving saline using an infrared thermometer from the start of the medical procedures. No medical procedures were performed on the naive (control) animals; they were exclusively given behavioral tests, although the layout of the cages allowed them to observe other animals. The design and details of the experiment are illustrated in Figure 1.

### 2.3. The “Confidence” Methodology

We propose a series of tests for training primates using the method of positive reinforcement. Three minutes were allotted for each test. If the experimenter left the room, the progress of the experiment was monitored using a camera (Xiaomi Yi 4 K Action Camera, Xiaomi Inc., Beijing, China) with a timer on a tripod, which was preinstalled in the testing room. Depending on the preferences of each primate, nuts or candied fruits were offered as a food reward in all tests. The order in which the animals were tested was randomized each time using random number generation. A refusal to participate was scored as 0 points. The test program timeline in detail is described in the Experimental Design section.

Procedure:

1. In the first stage, to initiate contact between the primate and the experimenter, it was necessary to approach the primate’s cage carefully, without startling the primate with sudden movements, and place a treat on the edge of the cage so that it would not fall off. The experimenter then exited the testing room or moved out of the primate’s line of sight. The primate needed to overcome stress and take the treat. If successful, a score of 1 point was awarded.
2. If the primate successfully completed Item 1, the next task was to take the treat from the edge of the cage in the presence of the experimenter. For this, all the actions of the previous item had to be repeated, but with the experimenter remaining in the testing room within the primate’s line of sight, placing the treat on the edge of the cage, and then stepping back a few meters and waiting. If successful, a score of 2 points was awarded.
3. In order to implement the next stage, a removable platform with a flat and non-slip surface was required, which was set up in front of the animal’s cage so that the primate could freely manipulate objects on its surface. After installing the platform, a treat was placed on it; then, the experimenter stepped back, and the primate had to take the treat. If successful, a score of 3 points was awarded.
4. In order to implement the following stage, a removable platform was also required. After setting up the platform, three cups were arranged on it in a row, and a treat was put in one of them; then, the experimenter exited the testing room or moved out of the primate’s line of sight. The test was considered successfully completed if the primate took the treat. If successful, a score of 4 points was awarded.
5. The next task for the primate was to take the treat from the platform in the presence of the experimenter. If successful, a score of 5 points was awarded.
6. The primate was required to complete Item 4, but in the presence of the experimenter. If successful, a score of 6 points was awarded.
7. The final stage was a task where the primate had to take a treat from the hand of the experimenter. For this, it was necessary to carefully approach the primate’s cage and extend the treat so that the primate could freely reach it. If successful, a score of 7 points was awarded.

### 2.4. Behavioral and Cognitive Tests

The training period for all tests was approximately 1 month and was initiated after the primates had completed the “Confidence” test and then, following the medical manipulations, the examination period began (Figure 1). For all tests in which there was a probability of random rather than deliberate choice, the primates were offered the chance to make several attempts in succession. All tests and our modernizations of the methods, as well as comments on them, can be found via the link in the Supplementary Materials section.

#### 2.4.1. Test 1: The “Tool Use and Properties” Battery—Study of Cognitive Abilities Based on the Primate Cognition Test Battery (PCTB)

The study of cognitive abilities was based on a PCTB-based cascade of tests [19]. Three minutes were allocated for each trial. It is important to note that primates are naturally curious and may be interested in an object as such. However, the study specifically aimed to investigate the primates’ ability to manipulate objects and understand their properties, i.e., to use tools to obtain bait. Thus, if a primate took an object but ignored the bait, it was considered that the test was not completed. 

**Figure 2:**
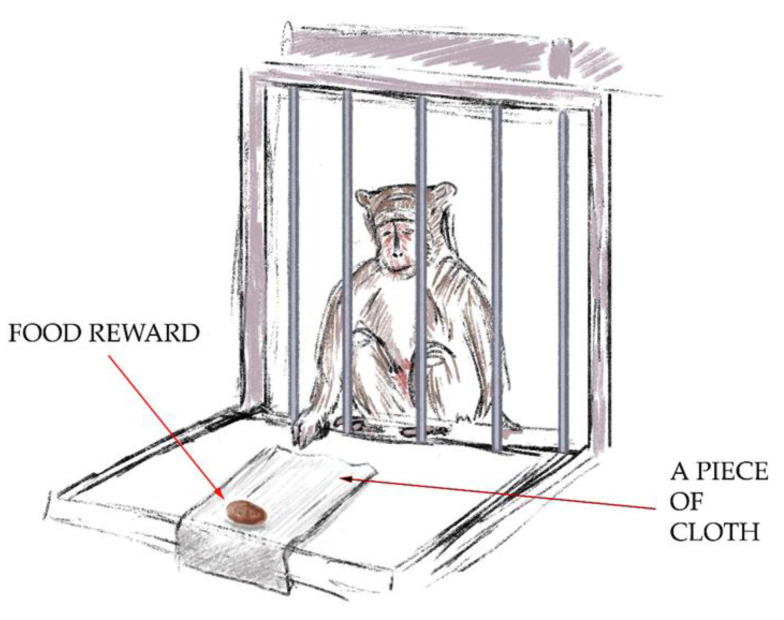
The “Tool Use: Pulling Cloth with Treat” test.

**Figure 3:**
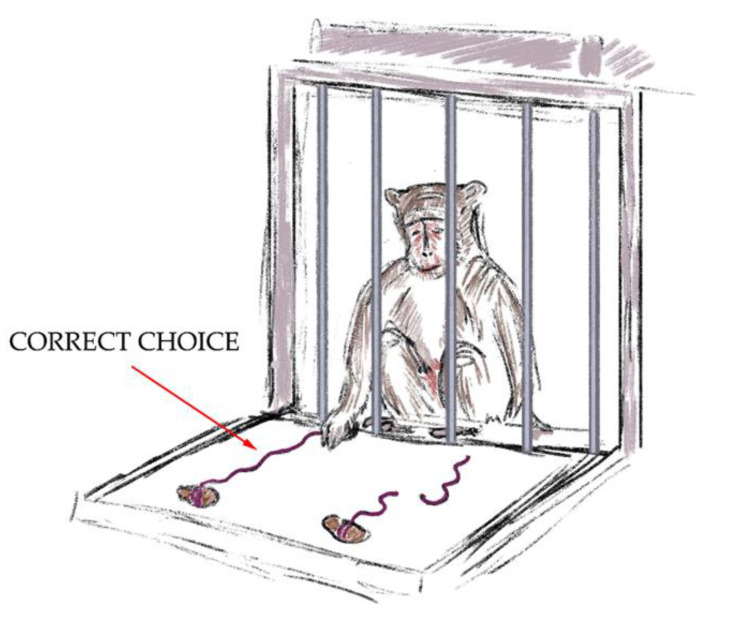
The “Tool Properties: Whole and Cut Threads with a Treat” test.

Procedure:

1. The “Three Cups” test. In this test, in front of each primate’s cage, three identical cups were posed in a row with a treat in one of them (random choice). The primate could easily see the treat. The aim of the test was for the primate to choose a cup with a treat from the first try. A successful outcome scored 1 point. A test was not successful when the primate chose a cup without a treat. This test is necessary for teaching object operation on the platform, with the aim of gradually increasing the complexity of the tasks. The test is similar to “Item 6” in the Confidence battery.
2. The “Tool Use: Pulling Cloth with Treat” test (Figure 2). For the second test, the primates had to get the treat by pulling a piece of cloth. A piece of cloth (30 cm × 15 cm) with a treat on its end was posed so that the primate could reach the cloth, but the treat was rather far away from the monkey, such that it could not grab the treat with its paw. The primate had to use the tool to get the treat with it. The aim of the test was to demonstrate the ability to manipulate objects outside the cage and understand the spatial relationship between objects. A successful outcome scored 2 points.

3. The “Tool Properties: Pulling a Thread with a Treat “ test. In this test, a thread of 15 cm was placed on the platform of the cage with the treat on its end. The primate had to pull the thread (use the tool) to get a treat, demonstrating the ability to manipulate objects outside the cage. A successful outcome scored 2 points.
4. The “Tool Properties: Whole and Cut Threads with a Treat” test (Figure 3). In this stage of the training, the primate had to choose one of two threads (15 cm). One thread was cut into two pieces and the other thread was whole. There were treats at the ends of both threads. The research objects were blocked from view by a barrier and the animal did not know on which thread each treat was. The primate could not reach the treats with its paw. After taking away the barrier, the primate could only get a treat by pulling the unbroken thread, demonstrating the ability to manipulate objects outside the cage and understand the properties of objects. A successful outcome scored 4 points. If the primate chose the wrong thread on the first attempt, the experiment was stopped. The threads were totally the same in terms of color, length, and material.

#### 2.4.2. Test 2: The “Memory” Battery—Study of Cognitive Abilities Based on PCTB

The study of cognitive abilities was based on a PCTB-based cascade of tests [19].

**Figure 4:**
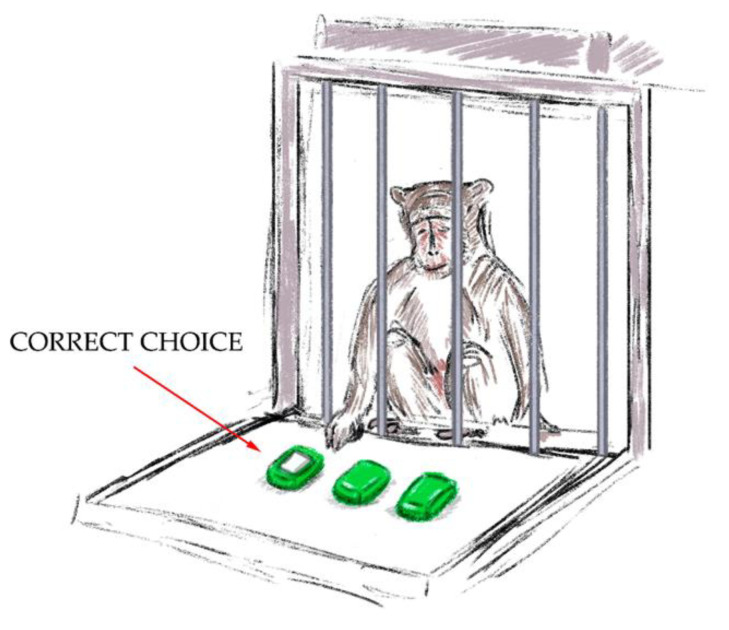
The “Sticker Cup” test.

Procedure:

1. The “Sticker Cup” test (Figure 4). There were three overturned cups put in a row on the platform in front of the testing cage. One of the cups had a white sticker on it. The experimenter showed the primate under which cup he put the treat and marked it with a white sticker. The aim of the experiment was to choose the right cup. A second choice was not given to the primate if it could not choose the right cup the first time. The experiment was successful when the primate chose the cup with the white sticker on the initial try. During the training sessions, it was crucial to solidify the primate’s connection between the sticker and the treat. A successful outcome was awarded 1 point.

2. The “Spatial Memory: 3 Upside Down Cups and 1 Treat” test. The experimenter prepared three identical cups and put them on a platform in a row in front of the cage. All the cups were overturned. A treat was placed under one of the cups (random choice) for the primate, which was shown to it. The animal could see the cups and had to choose the one with a treat under it. If the primate chose an empty cup, it did not have a second chance during the same experiment. A correct answer was accepted only on the first try. This demonstrated the capabilities of spatial memory and concentration on an object. A successful outcome was awarded 2 points.
3. The “Memory and Associations: Sticker Cup and a Barrier” test. This test was given to primates who could successfully pass the “Sticker Cup” test. Three overturned cups were put in a row in front of the cage and were hidden with a barrier. One of the cups had a white sticker on it. A treat was put under the cup marked by the sticker. As the primate already had experienced an experiment with cups and stickers, it was supposed to have formed and reinforced the association of the treat with the cup marked with a sticker. When the barrier was removed, the primate could choose the cup. In cases wherein the animal chose a cup without a white sticker, it was not allowed to make a second choice. The experiment was successful when the primate chose the cup with the sticker on the first try, thereby demonstrating a reinforced memory trace of the object. A successful outcome was awarded 4 points.

#### 2.4.3. Test 3: The “Box”—Research Activity

**Figure 5:**
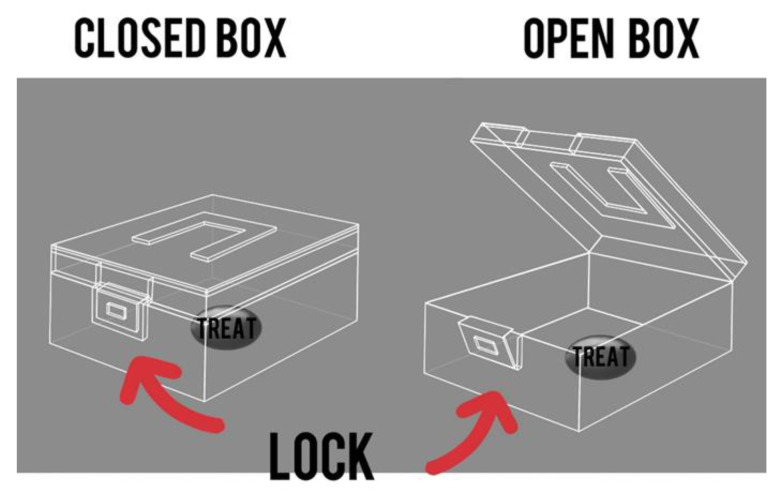
The “Box” test. Graphic design of the box and lock.

The “Box” test is a method designed to test the abilities of primates in solving intellectual and memory tasks. The primate must acquire the skill of unlocking a box to reach a treat inside. The tests were carried out both before (the training period) and after 5 days of medical manipulation to assess any differences in outcome. A 10 min timeframe was allotted to complete the task. The duration it took the primate to finish the task was documented, as well as any positive or negative changes in time. The primate must undergo training before the test, which should be conducted at least three times, depending on the success of each individual primate, with breaks ranging from several days to a week and 2–3 attempts at a time. The best training period result can serve as a baseline for assessment. The experimental process can be monitored with a camera on a tripod with a timer, which should be set up in the testing room in advance. Figure 5 showcases the graphical layout of the box.

#### 2.4.4. Test 4: The “Reaction to a New Object”—Research Activity

We conducted the evaluation of the research activity by employing our adapted version of the “Reaction to a New Object” methodology [21]. Before conducting experimental manipulations, the primates were trained by being shown object A three times (A1, A2, and A3) to become familiar with it and form memories related to interacting with this object. Object A was presented three times: on the first day, then 10 days later, and finally the day after that. A few days later, on the third day after the saline injection and blood sampling of two animals, object A was introduced alongside a novel object (B (B1)) that had not been known to the subjects before. The conventions for the test are outlined in Table 1.

**Figure 6:**
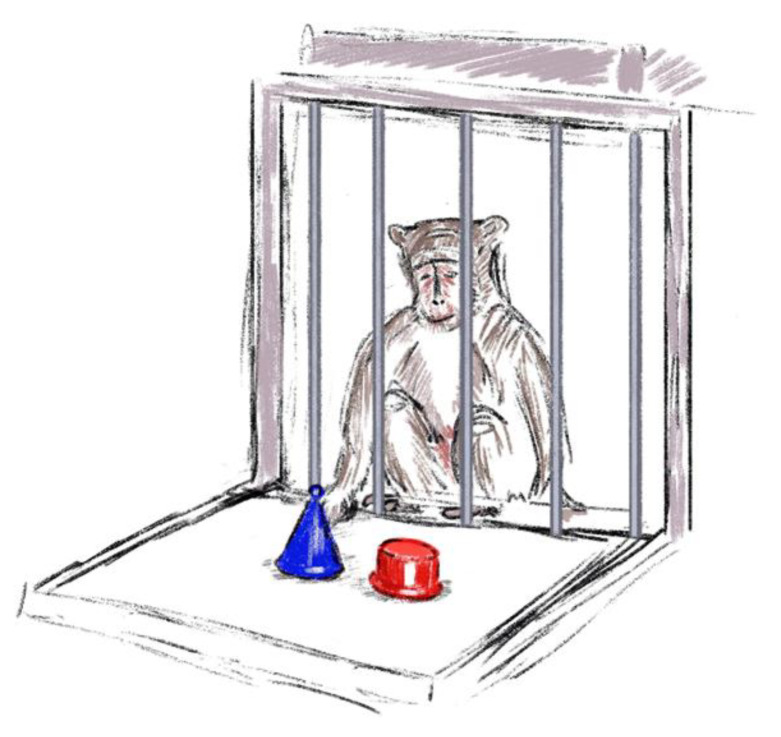
The “Reaction to a New Object” test.

The animal was given an object featuring novelty as its key characteristic. A plastic red cylinder measuring 6 × 6.5 cm (offered for study in a training period) and a plastic blue cone measuring 10 × 6.5 cm (offered after manipulations together with the red cylinder as a new item) were used as objects (Figure 6). Every primate had 300 s to study the object carefully. The video camera, which provided the possibility to observe and study the behavior of primates, was installed in the room. When the animal stopped making contact with the object, the time was marked as "left". Three important activities were studied during the experiment: reaction latency, defined as the period from the initiation of the test to the first instance of contact; activity, defined as the summation of the time intervals during which animals interacted with the object; concentration, defined as the mean value of the time intervals during which animals interacted with the object.

#### 2.4.5. Test 5: The “Tiredness” Battery—Tiredness and Performance Capacity

The cognitive abilities of each monkey were evaluated through a series of tests, as discussed in our work. We did not include test 4, “Reaction to a New Object”, in the “Tiredness” cascade; only Tests 1, 2, 3 were included. Test 4 was offered on day 3 to further assess this period and to not complicate the program of the study on day 5. We assessed tiredness by analyzing the quantity of tests and attempts that were successfully completed. The animals took the tests consecutively, until each primate declined to work, became disinterested, or committed three or more consecutive mistakes. At that point, the primates stopped performing tasks and their scores were tallied. All tests were followed in sequential order with three trials for each individual test. Each primate was already familiar with the order and types of trials. The scores achieved on the final day of training for the specific tests were established as the baseline results before any medical procedures were carried out. Then, the test was offered again on the 5th day after the start of the medical manipulations.

### 2.5. Statistical Analysis

In order to identify the connections between the scores in the Confidence test and success in behavioral test tasks, Kendall’s rank correlation, τ, was calculated. The research on the influence of cage location on the confidence in the experimenter and the research activity of the primates, as well as a comparison of primates by sex in behavioral tests, was conducted using the Kruskal–Wallis criterion. For multiple post hoc comparisons, Dunn’s method was used. Primates were compared by sex in the Confidence test using the Mann–Whitney test. The calculations were executed utilizing the PAST software package (v.3.19). GraphPad Prism 9.0.0 (GraphPad Software) and the PAST software package (v.3.19) were used to perform the data visualization. 

## 3. Results

### 3.1. The Confidence Methodology

**Figure 7:**
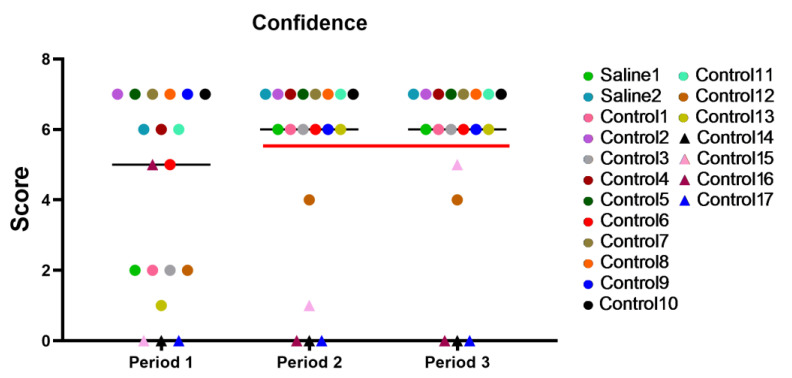
Individual results with time for all primates during different periods of the 2-week training. The red line separates the functional primates (above 6 points) from the non-functional ones (below 6 points) for the second and third training periods. Saline had not yet been administered to Saline 1 and Saline 2 primates during the training period. The primates without manipulations were labeled Control 1–17.

The graph depicted in Figure 7 shows the results with time for all the primates during different periods of the 2-week training period. Period 1: the first day of testing; Period 2: the results at the end of the first week of training; Period 3: the last day of training. After Period 2, a significant performance gap could be observed between the capable primates and those who had difficulties trusting the experimenters and struggled with task performance over time. Five primates (C12, C14, C15, C16, and C17), approximately 26%, were unable to perform the tasks and interact with the experimenter. 

### 3.2. Behavioral and Cognitive Tests

After a 1-month training period of behavioral tests, blood samples were collected from two primates (S1 and S2) on the first day of the experiment (day 0); then, also on day 0, the primates were administered a sterile saline solution; additionally, the temperature was monitored daily with an infrared thermometer right from the start of the medical procedures. On days 2 and 4 of the experiment, the experimenters also took blood samples. A total of 17 monkeys (C1–C17) were not subjected to any medical manipulations and solely underwent tests to assess their cognitive abilities. Cognitive tests were conducted on days 3 and 5 after the administration of the saline solution and the first blood sampling in the experimental group.

#### 3.2.1. Test 1: The “Tool Use and Properties” Battery—Study of Cognitive Abilities Based on the Primate Cognition Test Battery (PCTB)

**Figure 8:**
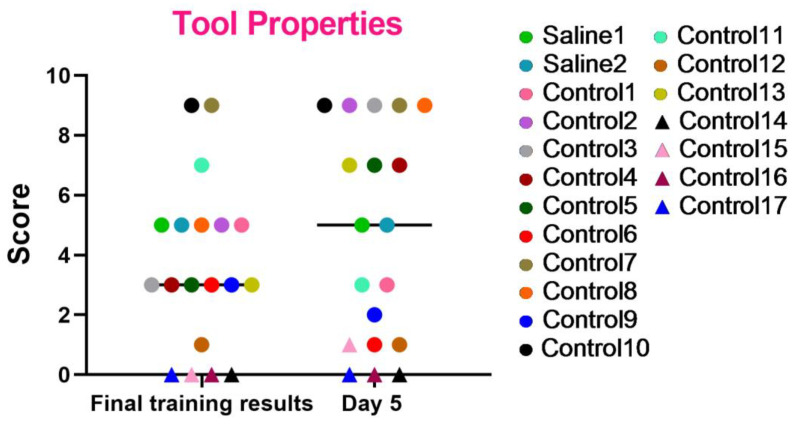
The outcomes of the “Tool Use and Properties” test in relation to time during the final day of the training and on day 5 after the manipulations. Saline had not yet been administered to the Saline 1 and Saline 2 primates during the training period; homonymy was retained throughout for ease of reference. The primates without manipulations were labeled Control 1–17.

These series of tests were created to evaluate the essential abilities required for primates to operate effectively within their natural habitats. The results, as shown in the graph (Figure 8), indicate that most primates successfully completed the tasks and improved their performance over time. However, primates that initially struggled with the Confidence test and scored low (C12, C14, C15, C16, and C17) or even refused to work with experimenters also failed to perform well in this test or scored the lowest relative to other primates. Primates C1, C6, and C9 also showed a decrease in their scores, which could be related to stress. The test results also demonstrate that two primates subjected to medical procedures (blood collection and saline injection) maintained their previous level of results and did not decrease their scores. Thus, it can be concluded that medical procedures did not affect their ability to understand object characteristics and interact with them.

#### 3.2.2. Test 2: The “Memory” Battery—Study of Cognitive Abilities Based on PCTB

**Figure 9:**
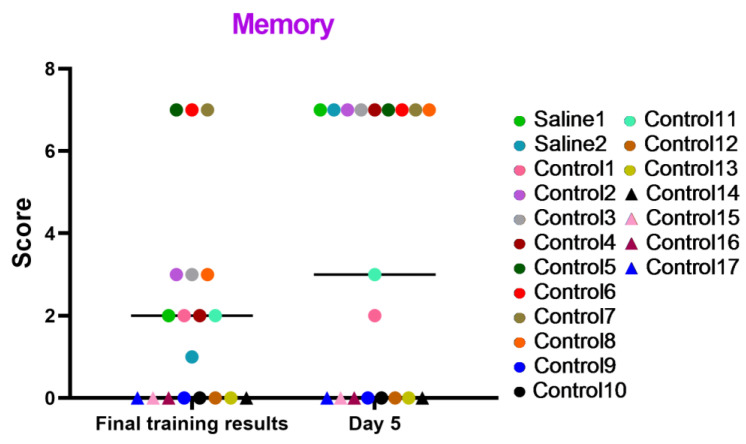
The results of the “Memory” test in relation to time during the final day of the training and on day 5 after the manipulations. Saline had not yet been administered to the Saline 1 and Saline 2 primates during the training period; homonymy was retained throughout for ease of reference. The primates without manipulations were labeled Control 1–17.

These tests focused on evaluating the memory status and cognitive processes of manipulating, associating, and storing acquired information of the primates under study. The results displayed in the graph (Figure 9) indicate that the majority of the primates successfully coped with the tasks and improved their performance over time. The primates that initially had difficulties with the Confidence test and scored low (C12, C14, C15, C16, and C17) or outright refused to work with experimenters also failed to perform well in this test. Additionally, we saw several other primates that failed the task (C9, C10, and C13), which could be related to stress or the relative difficulty of the test itself, which required maximum engagement, attentional focus, and intelligence from the primates. Moreover, the results in the “Memory” test show that two primates subjected to medical manipulations (blood collection and saline injection) improved their results on day 5 of the study. The control primates, for the most part, also scored higher. Thus, it can be concluded that medical manipulations do not affect the mnemonic functions of primates.

#### 3.2.3. Test 3: The “Box”—Research Activity

**Figure 10:**
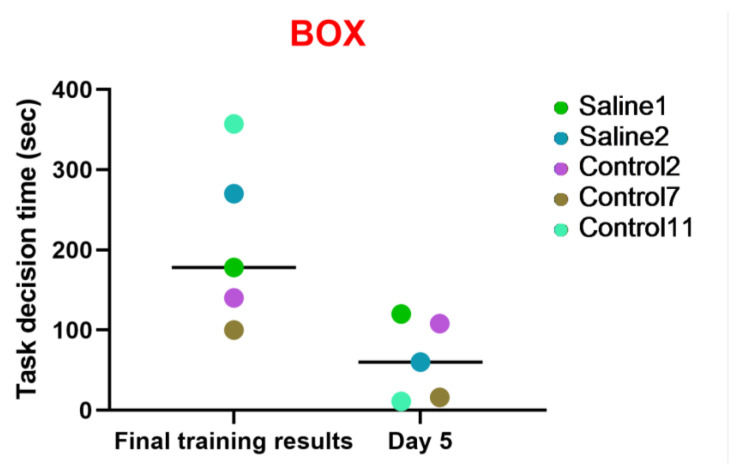
The results of the “Box” test in relation to time during the final day of the training and on day 5 after the manipulations. Saline had not yet been administered to the Saline 1 and Saline 2 primates during the training period; homonymy was retained throughout for ease of reference. The primates without manipulations were labeled Control 1–17.

The aim of this examination was to evaluate the subject primates’ proficiency and quickness in solving intellectual and memory-based tasks related to previously learned skills and the dynamics of the outcomes. The time required for the subject to solve the task was recorded, along with either positive or negative temporal dynamics. The five primates (S1, S2, C2, C7, C11) were offered to undergo testing at the training stage; they successfully completed the task and were admitted to the fifth day of the study. All these primates showed positive performance dynamics. The results obtained in the “Box” test also indicate that two primates subjected to medical manipulations (blood collection and saline injection) in a similar way to the control group improved their performance and coped well with the task. All the primates reduced the time taken to solve the test. Thus, it can be concluded that medical manipulations do not affect the ability to solve intellectual tasks. These data are presented in Figure 10.

#### 3.2.4. Test 4: The “Reaction to a New Object”—Research Activity

This test was carried out before manipulations (training period results) and on day 3, after the start of medical procedures, and was designed to measure the latency of reaction, activity level, and concentration of attention in animals. The results of the study were presented in numerical values (parameters: “latency”, “activity”, and “concentration”).

**Figure 11:**
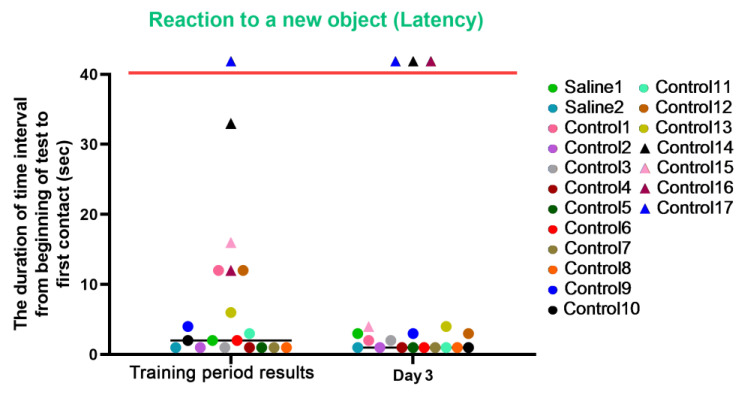
The results of the latency characteristic evaluation in the test “Reaction to a New Object” in terms of dynamics before manipulations (training period results) and on day 3 after manipulations. Primates that were unsuccessful in the test and declined to engage with the experimenters or whose test completion time exceeded 100 s are situated above the red line. Saline had not yet been administered to the Saline 1 and Saline 2 primates during the training period; homonymy was retained throughout for ease of reference. The primates without manipulations were labeled Control 1–17.

For the latency characteristic, we observed an improvement in reaction speed in most control primates on day 3 after the procedures. The primates who initially had difficulties in the Confidence test refused to interact with the experimenters (C14 and C17) or significantly worsened their reaction speed and struggled with overcoming stress (C16) on day 3 of the experiment. In the group of primates with medical manipulations, we did not observe a deterioration in indicators; both primates maintained their previous indicators and good reaction speed. The results are presented in Figure 11.

**Figure 12:**
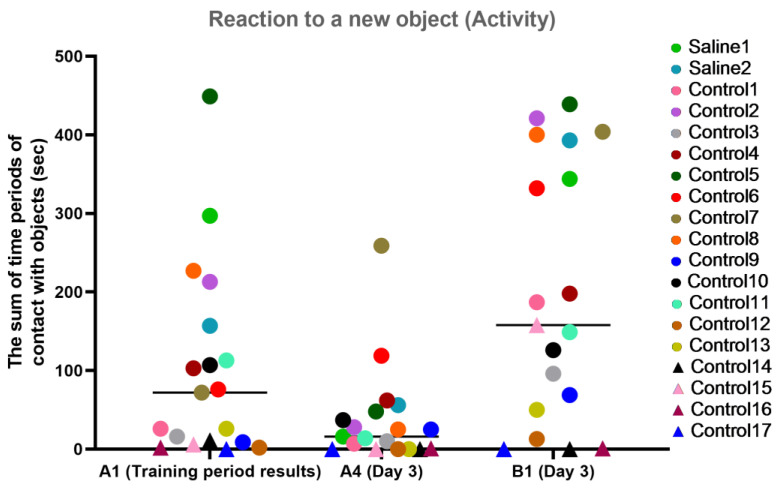
The results of the activity characteristic evaluation from the test “Reaction to a New Object” in terms of dynamics before manipulations (A1 (training period results)) and on day 3 after manipulations (A4 and B1). Saline had not yet been administered to the Saline 1 and Saline 2 primates during the training period; homonymy was retained throughout for ease of reference. Primates without manipulations were labeled Control 1–17.

For the activity characteristic, most primates from the experimental group showed a healthy interest in the new object (B1) and held onto the memory of an object they had previously been familiar with (the difference between A1 and A4). The primates who initially had difficulties in the Confidence test (C12, C14, C15, C16, and C17) refused to interact with the experimenters or had weak interest in the objects, showing low activity on day 3 of the experiment. The primates with medical manipulations had good activity indicators. The results are presented in Figure 12.

**Figure 13:**
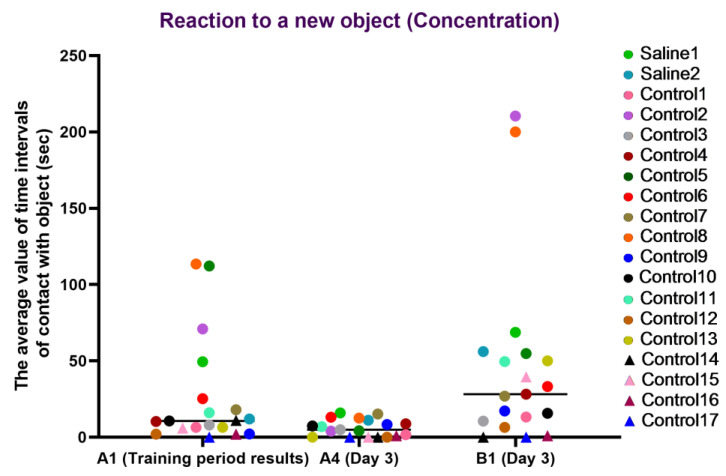
The results of the concentration characteristic evaluation in the test “Reaction to a New Object” in terms of dynamics before manipulations (A1 (training period results)) and on day 3 after manipulations (A4 and B1). Saline had not yet been administered to the Saline 1 and Saline 2 primates during the training period; homonymy was retained throughout for ease of reference. Primates without manipulations were labeled Control 1–17.

For the concentration characteristic, a healthy concentration on the new object (B1) was observed in most primates from the experimental group, whereas, in most primates, concentration on the old item (A4) decreased, which may indicate a retained memory of the previously familiar object. The primates who initially had difficulties in the Confidence test (C12, C14, C15, C16, and C17) refused to interact with the experimenters or had weak interest in the objects, showing low activity on day 3 of the experiment. During medical manipulation with one primate, we observed a small difference between A1 and A4, and the difference between A4 and B1 was good, which, overall, may indicate a healthy interest in new objects and good concentration but a poor memory of the old object; however, it is difficult to make definitive conclusions due to the small sample size. The second primate with medical manipulations exhibited good concentration on the new object and retained the memory of the old object. The results are presented in Figure 13.

#### 3.2.5. Test 5: The “Tiredness” Battery—Tiredness and Performance Capacity

**Figure 14:**
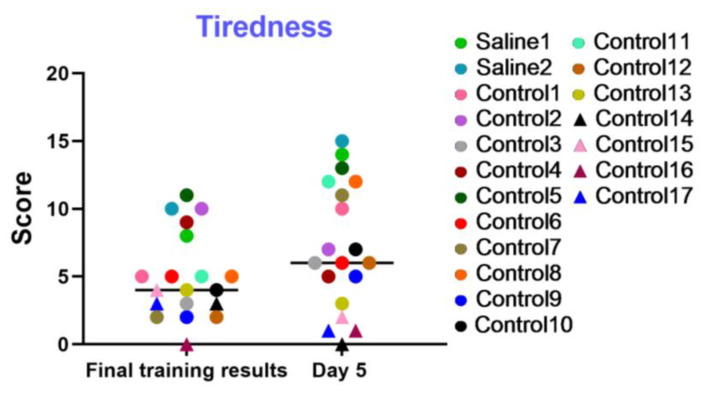
The results of the “Tiredness” test in terms of dynamics on the last day of training (final training results) and on day 5 after manipulations. Saline had not yet been administered to the Saline 1 and Saline 2 primates during the training period; homonymy was retained throughout for ease of reference. Primates without manipulations were labeled Control 1–17.

This examination was utilized to dynamically evaluate the level of fatigue, the general psychophysiological condition, and the motivation of the test primates during training sessions and on day 5 of the experiment. The results displayed in the graph (Figure 14) indicate that most primates successfully coped with the tasks and improved their performance over time. The primates who initially had difficulties in the Confidence test and scored low, or even refused to work with the experimenters, scored the lowest in this test (not more than 4 points). Negative dynamics were shown by primates C13, C14, C15, C16, and C17. Primates C3, C7, C9, C10, and C12 scored low in the final training results but increased their scores over time. Additionally, the results of the “Tiredness” test show that two primates who were subjected to medical manipulations improved their performance and coped well with the tasks presented. Thus, it can be concluded that medical manipulations do not affect the performance capacity and tiredness of primates.

### 3.3. Searching for a Correlation between Successful Completion of the Confidence Test Tasks and Subsequent Success in Behavioral Tests

In order to identify the relation between the scores in the “Confidence” test and success in behavioral test tasks, Kendall’s rank correlation, τ, was calculated. Relationships were deemed statistically significant if *p* ≤ 0.05, insignificant if *p* > 1, and instances of 0.05 < *p* ≤ 1 were deemed intermediate cases, where the tendency towards relationships was discussed. The calculations were performed using the PAST software package (v.3.19). “Period 1” in the tests referred to the initial results in the “Confidence” test obtained during the first half of the learning phase. “Period 2” in the tests referred to results obtained during the second half of the learning phase. “Period 3” in the tests also referred to the results obtained during the second half of the learning phase. The scores in behavioral tests were understood as the total score for some of the most indicative tests in which primates participated (total test scores: “Tool Use and Properties”, “Memory”, and “Tiredness”). A positive and statistically moderate significance relation was found between trust scores and a higher level of passing behavioral tests. For Period 3, the relationship was more pronounced than for the preceding time periods. Kendall’s correlation coefficients and their significance levels are presented in Table 2.

### 3.4. The Influence of Cage Location on Confidence in the Experimenter and the Research Activity of Primates

**Figure 15:**
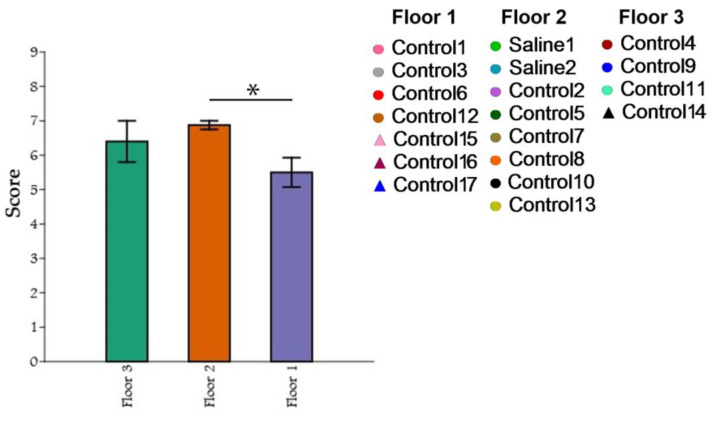
The influence of cage location on confidence in the experimenter and the research activity of primates. Floor 1: cage location at leg level; Floor 2: cage location at waist level; Floor 3: cage location at head level. Asterisks indicate statistically significant differences.

A comparison of the three groups of primates based on the maximum results of the “Confidence” test with a non-normal distribution was conducted using the Kruskal–Wallis criterion. For multiple post hoc comparisons, Dunn’s method was used. Statistical significance was determined at *p* ≤ 0.05, while *p* ≥ 0.10 indicated a lack of significance. For cases falling within the range of 0.05 ≤ *p* ≤ 0.10, potential trends towards differences were examined. The PAST software package (v.3.19) was used to perform the graphical depictions and calculations. During the comparison of the three groups of primates using the Kruskal–Wallis method, statistically significant differences were found in the number of maximum scores obtained in the “Confidence” test (H = 6.003; *p* = 0.02309). The post hoc comparisons using Dunn’s method showed that the groups differed significantly. The intergroup differences were primarily due to the differences between the primates located on the first and second floors (*p* = 0.006584), as well as a tendency towards significant differences between the first and third floors (*p* = 0.1215) (Figure 15). Significant differences between the second and third floors were not found.

### 3.5. Comparison of Primates by Sex in Behavioral Tests and in the Confidence Test

We compared the males and females only on the second and third floors, due to a notable majority of males presented on the first floor, which made analysis impossible. The comparison of males and females across different floors was conducted according to the maximum results obtained from the “Confidence” test over the entire training period using the Kruskal–Wallis criterion. For multiple post hoc comparisons, Dunn’s method was used. Statistical significance was determined at *p* ≤ 0.05, while *p* ≥ 0.10 indicated a lack of significance. For cases falling within the range of 0.05 ≤ *p* ≤ 0.10, potential trends towards differences were examined. During the comparison of the primate data using the Kruskal–Wallis method, statistically insignificant differences were found in the number of maximum points scored in the “Confidence” test (H = 1.093; *p* = 0.7786). The post hoc comparisons using Dunn’s method showed that the groups of females and males on the third and second floors differ insignificantly (Table 3).

The Mann–Whitney test was used to compare males and females. The comparison was considered statistically significant at *p* ≤ 0.05. In analyzing the test outcomes, no statistically significant variations were detected in the behavioral tests among primates of varying sexes. The outcomes are presented in Table 4.

## 4. Discussion

The aim of our research was the development and optimization of methodologies and the meticulous selection of tests to assess the behavioral responses and cognitive abilities of non-human primates. This also involved taking into account the complex and specific conditions of the virology laboratory for further work with neurotropic viruses and the adaption of all methodologies to the existing features. The primary requirements for such methods were low in terms of labor intensity, despite being informative.

Studies on the effects of neurotropic viral infections, which impact human higher nervous functions, have uncovered notable alterations in mental state and behavior [24,25]. Similarly, severe central nervous system (CNS) damage has been described due to acute neurotropic infections in primates [26,27]. During our experiment, we endeavored to select sensitive tests to detect even the most subtle changes in primate behavior. 

We present a series of tasks from the “Tool Use and Properties” cascade as an effective method for assessing primates’ physical cognition skills; how primates understand objects and their properties and the spatial and causal relation between objects. Our methodology includes the “Three Cups” test, which is not present in the original battery of tests that we referenced. This test is used to prepare primates for working with the platform and helps focus the primates on interacting with the experimenter and objects. Additionally, our version includes a scoring system to evaluate successful attempts, whereas the original battery of tests merely records correct and incorrect responses. We believe that our approach will facilitate the analysis of results. Our version is more simplified and focuses on the key aspects of tool use and its properties, while the original battery of tests includes more diverse and detailed tasks. We have selected the simplest tests for long-tailed macaques, which nonetheless help evaluate the behavioral aspect of interest to the researchers.

A series of tasks from the “Memory” cascade are presented as an effective method for assessing the mnemonic functions of primates. We have added two new tests based on the existing “Sticker Cup” and “Memory and Associations: Sticker Cup and a Barrier” tests. Additionally, in the “Spatial Memory: 3 Upside Down Cups and 1 Treat” test, we have slightly increased the difficulty by leaving only one treat, whereas the original method has two. Using a single treat allows for a more accurate assessment of the primate’s attention and memory, as well as reducing the chance of random selection. Our battery includes the use of a sticker to mark one of the cups, adding an element of association with a visual marker, which is absent in the original battery. The original PCTB includes tests with simple and double cup movements, allowing for the assessment of primates’ ability to track object movement, but this approach is much more complex for the primates. Instead, our battery includes tests aimed at assessing the primates’ ability to remember the location of the reward under a specific cup without movements. Our battery’s tests also include a learning element, wherein the primates need to associate the sticker with the reward, which is reinforced during training. The original battery does not have such an explicit training and reinforcement stage. Additionally, our version includes a scoring system to evaluate successful attempts to facilitate analysis.

The “Box” test was demonstrated to be an effective method to evaluate the skills of primates to solve intellectual tasks that require concentration, resourcefulness, and the preserved memory of interaction with an object. Based on previous work wherein a primate needed to retrieve a lure from a box using a tool [22], we have proposed our simplified version for long-tailed macaques.

We have shown that, by using our upgraded methodology [21], “Reaction to a New Object”, it is possible to study the exploratory activity of primates. This test allows for the assessment of reaction latency, activity, and attentional focus in primates, as well as the identification of mnemonic disorders and the evaluation of primates’ natural inclination towards novelty and knowledge. Regarding the latency characteristic, we observed an improvement in reaction speed in most primates. However, in the group of primates that were subjected to medical manipulation, we observed a slight decrease in indicators in one subject, while in another group, they remained at the previous level. Due to the small sample size, it is difficult to make definitive conclusions, but it can be supposed that medical interventions do not significantly affect the reaction speed of primates. Our methodology includes pre-training and repeated presentations of an object, which allows the animals to familiarize themselves with the object before the main test, and we can assess the dynamics of exploratory activity at several stages. This also allows us to compare the results before and after manipulation. The original methodology includes additional parameters such as contact type and more in-depth observation of primate behavioral patterns, whereas we include only the basic parameters (reaction latency, activity, and concentration).

The scientific literature describes numerous cases of patients complaining about increased tiredness and inefficiency after suffering infections [23,28,29]. In our work, we demonstrated that the “Tiredness” test battery can be used to evaluate the efficiency and tiredness of primates. Our approach is based on assessing performance dynamics using a comprehensive cascade of cognitive tests included in this article. This method is also well known thanks to the works of other authors [20]. Our method is convenient because it allows for the additional assessment of the tiredness parameter without the need for extra tests, which simplifies the work for experimenters. Moreover, it does not overload the primate’s program and does not involve any medical procedures (no motor endurance tests or physiological measurements are used), thus minimizing subjectivity (without behavioral observations).

By using our proposed cognitive–behavioral test program, the cognitive capabilities of primates can be assessed. Thus, it is possible to assess the impact of infectious load and immune response on the parameters under investigation.

Virological studies also require a sufficiently large number of analyses and medical procedures on a research subject. Therefore, the next important step in our work was to determine whether medical procedures, such as blood collection and injections, affect the cognitive abilities, behavior, and overall psychophysiological state of primates. To this end, we allowed all primates to undergo a series of tests and cascades of neurocognitive tests. The test results showed that medical manipulations do not significantly affect the behavior and cognitive abilities of the primates.

Most of the proposed cognitive tests seem accessible for long-tailed macaques and also cover a wide range of functions for the prospective study of CNS pathologies caused by various infections, immune responses, and drugs.

In our tests, we observed the individual dynamics of primates’ results in separate cognitive–behavioral tests; we noticed that some test monkeys experienced difficulties in some tests but showed good performance in other tests when considering the same or a related parameter. This could be related to stress reactions as well as to the individual personality traits of each primate. Based on the data from our research, we propose using a multi-faceted approach when selecting behavioral tests. This implies including several tests to analyze each parameter of interest, which will allow for a more complete and accurate representation of the behavioral characteristics of primates.

In our work, we present an upgraded methodology that facilitates the effective training of long-tailed macaques using positive reinforcement training (PRT), which has been successfully applied by other researchers when previously working with primates. The core principle of this methodology involves the systematic and gradual training of primates using positive reinforcement along with specifically designed tasks and exercises. Special attention was given to establishing trust between the experimenter and the animal, as well as creating a stimulating and safe environment for training.

We encountered significant behavioral differences between the animals in their abilities to solve complex tasks and interact with experimenters, which may be associated with increased vigilance and anxiety. This prompted us to introduce the “Confidence” test into the program. We also included nonperforming primates in the analysis to evaluate the dynamics of their results. Further analysis showed that the position of the cage could also affect the behavior of the primates.

By using the “Confidence” method, we explored whether the positioning of the cage relative to humans affects the primates’ trust in the experimenter and their research activity. Among three different configurations (the cage positioned at leg level, waist level, and head level), it was shown that the best positions for productive work and the primates’ trust in the experimenter are at waist level and head level, in order of priority. Primates in cages at leg level scored the lowest, suggesting that this configuration is not recommended for further research. When at this level, primates are less inclined to interact with the experimenter, reluctant to take treats, and struggle with any intellectual tasks presented in the experiment, preferring to spend most of their time at the back wall of the cage. This is likely due to poor visibility within the cage, as well as the animal’s difficulty in observing the experimenter and predicting their movements, causing stress and fear in the animal.

Some studies have indicated a difference in the intellectual capabilities of primates depending on gender [30,31]. Research on the impact of viral infection of the nervous system on behavior and cognitive abilities supposes that some important features carry gender differences, such as hormonal status, severity of illness, sensitivity to the virus, etc. These data necessitate the testing of both males and females to cover a multitude of aspects related to viral load. Our task was to determine whether gender differences affect the success rate in test performance and achievement using the “Confidence” methodology, also considering the location of the primate’s cage. We found no significant statistical differences between genders on different floors, indicating equal opportunities, in general, for both male and female long-tailed macaques in the experiment.

We determined that the “Confidence” methodology can also be used to make a qualitative selection of working primates into an experimental group. As early as Period 2, it was possible to select working primates into the group. Five primates (approximately 26%) were unable to perform tasks and interact with the experimenter; they also did not succeed in passing the cognitive tests or refused any interactions with experimenters altogether. There was a correlation between the scores in the “Confidence” test and successful completion of cognitive tests already at the early stages of testing (Period 1 and Period 3). Excluding such primates during the training period will be a good strategy that will help form a group of working primates, significantly save time, and support the obtainment of reliable results in experiments.

Our test program was previously validated in work [23]. Using these tests, we were able to detect some cognitive impairment in primates with inapparent orthoflavivirus infections.

## 5. Conclusions

1. Overall, the proposed upgraded methodologies provide a robust framework for the comprehensive assessment of primate cognition and behavior. These methods facilitate the effective training of long-tailed macaques, ensuring reliable and meaningful experimental results.
2. The “Confidence” methodology can be used to make a qualitative selection of working primates into an experimental group.
3. The simplified version of the “Tool Use and Properties” cascade proved its effectiveness in assessing primates’ understanding of objects and their properties and their spatial and causal relations.
4. The enhanced “Memory” cascade provided an accurate assessment of primates’ memory and attention.
5. The “Box” test was demonstrated to be an effective method to evaluate the skills of primates used to solve intellectual tasks.
6. The upgraded “Reaction to a New Object” methodology enabled the study of primates’ exploratory activity, reaction latency, and attentional focus.
7. The “Tiredness” test evaluates primates’ efficiency and tiredness without additional tests or medical procedures.
8. Routine medical procedures did not significantly affect the cognitive abilities or behavior of long-tailed macaques.
9. Cage positioning affects primates’ research activity and trust in the experimenter.
10. No significant statistical differences were found between male and female primates in the proposed tests of cognitive performance.

## Figures and Tables

**Table 1:** Conventions for the “Reaction to a New Object” test.

Before Manipulations	Day 3 Post-Manipulations’ Start
A1—The initial exposition of object A (red cylinder), at the beginning of the training period	B1—The initial exposition of object B (blue cone), which was presented as a novel object after medical manipulations, with the object that was previously known (A4) (red cylinder; the fourth exposition of object A)
A2—The second exposition of object A (red cylinder), 10 days after the exposition of object A1	
A3—The third exposition of object A (red cylinder), the day after the exposition of object A2	

**Table 2:** Correlations between the successful completion of the Confidence test tasks and further success in behavioral tests. Statistically significant differences are marked with an asterisk.

	Correlation Coefficient (τ)	Significance Level (*p*)
Confidence Period 1: total score in behavioral tests	0.50594	0.0024714 *
Confidence Period 2: total score in behavioral tests	0.5773	0.00054767 *
Confidence Period 3: total score in behavioral tests	0.82009	0.00044573 *

**Table 3:** *p*-Values obtained from comparing males and females on various floors in the Confidence test.

	Male Floor 3	Female Floor 3	Male Floor 2	Female Floor 2
Male floor 3		0.3102	0.7234	0.8891
Female floor 3	0.3102		0.48	0.383
Male floor 2	0.7234	0.48		0.8387
Female floor 2	0.8891	0.383	0.8387	

**Table 4:** A comparison of primates by sex in the behavioral tests.

	Memory		Tool Use and Properties		Tiredness	
	U-Statistic	*p*-Value	U-Statistic	*p*-Value	U-Statistic	*p*-Value
Males (*n* = 10) vs. females (*n* = 9)	40.5	0.6076	30	0.22768	38	0.59185

## Data Availability

Original materials presented in the research are included in the article; further inquiries may be directed to the corresponding author.

## Supplementary Materials

The following supporting information can be downloaded at https://www.mdpi.com/article/10.3390/brainsci14070635/s1, Table S1: Supporting information about the methods of the behavioral and cognitive Tests.
