# Supplementary material for: An Integrative Approach to the Study of Cognitive Abilities in a Non-Human Primate Model in a Virology Laboratory Environment
Source: Brain Sci. 2024 Jun 26;14(7):635. doi: 10.3390/brainsci14070635 (PMC11274874; doi:10.3390/brainsci14070635)
Supplement: Supplementary file 1 [file brainsci-14-00635-s001.zip › brainsci-3016144-supplementary.pdf]

Supplementary Table S1. Supporting Information about Methods of the Behavioral and Cognitive Tests.

№	Method name	Original Method	The method modified by us	Link to the original method
I. Study of Cognitive Abilities Based on the Primate Cognition Test Battery (PCTB). The "Tool Use and Properties" Battery.				
1.	Tool Use	A reward was placed on a table approximately 20 cm out of reach of the subject in front of their cage. A wooden stick (20 cm in length) was provided for the subject. In this experiment no plastic panel was used. The animals could handle the stick through the wire mesh of their cage. To be successful the subject had to use the tool to retrieve the out of reach object or food within two to three minutes. A correct response was scored if the subject was able to retrieve the reward.	**Our point 3.** The "Tool Properties: Pulling a Thread with a Treat " test. The experimenter placed one thread (15 cm) on the platform, with the treat tied to the far end of the thread and out of the primate's reach. The primate needed to use the tool (thread) to obtain the treat, thereby demonstrating the ability to manipulate objects outside the cage. A successful outcome scored 2 points.	Herrmann, E.; Call, J.; Hernàndez-Lloreda, M.V.; Hare, B.; Tomasello, M. Humans Have Evolved Specialized Skills of Social Cognition: The Cultural Intelligence Hypothesis. Science 2007, 317, 1360–1366, doi:10.1126/science.1146282.<br>Additional materials:<br>https://www.researchgate.net/publication/293979708_File_S1/fulltext/
2.	Tool Properties	**Side:** The experimenter put two identical pieces of cloth (15 cm x10 cm) on the platform behind an occluder and placed a reward on top of one piece of cloth, whereas the other reward was placed directly next to the other cloth piece (i.e. making the second tool ineffective for retrieving the food). After the occluder was removed, the subject could only retrieve the reward by pulling the piece of cloth with the reward on top of it.	**Our point 2.** The "Tool Use: Pulling Cloth with Treat" test. The experimenter set up a platform, laid a piece of cloth (30 cm x 15 cm) on it, and placed a treat on top of the cloth so that the monkey could reach the cloth but could not grab the treat with its paw. The primate needed to use the tool (cloth) to obtain the treat, thereby demonstrating the ability to manipulate objects outside the cage and understanding the spatial relationship between objects. A successful outcome scored 2 points.	
		**Broken wool:** The experimenter put up an occluder and placed two strings of wool on the platform, one of them was cut into two pieces. Like in the Ripped cloth condition both strings were arranged in a way that the gap was visible, but that both resulted in an equal	**Our point 4.** The "Tool Properties: Whole and Cut Threads with a Treat" test. The experimenter set up a barrier and placed two threads (15 cm) on the platform. One of the threads was cut into two pieces. Treats were tied to the far end of the threads	

		length. A peanut was tied to the far end of the wool strings out of the subject's reach. After removing the occluder, the reward could only be retrieved by pulling the intact piece of wool.	and out of the primate's reach. After removing the barrier, the primate could only obtain the treat if it pulled the intact piece of the thread, thereby demonstrating the ability to manipulate objects outside the cage and under-stand the properties of objects. A successful outcome scored 4 points. If the primate pulled on the torn thread first, the test would be stopped. Both threads were identical in terms of length, color, and material.	56bd3f0708aed695994639f5/293979708_File_S1.pdf
**The main differences:**		1. "Three Cups":<br>Our version includes the "Three Cups" test, which is not in the original battery of tests. This test is used to prepare primates for the platform and in helps focus primates to work with the experimenter and objects.<br>2. Scoring System:<br>Our version incorporates a scoring system to evaluate successful attempts, while the original test battery simply records correct and incorrect responses. We believe that our approach will make it easier to analyze the results.<br>3. Detailing:<br>Our version is more simplified and focuses on key aspects of tool usage and tool properties, while the original test battery includes more varied and detailed tasks. We have selected the simplest tests for long-tailed macaques, which nevertheless help to assess the behavioral aspects that are of interest to the researchers. The original battery of tests includes dozens of tasks. We have reduced the number of tasks and selected the most appropriate ones to identify the effects of interest.		
II. Study of Cognitive Abilities Based on PCTB. The "Memory" Battery				

<table>
<tr><td>1.</td><td>Spatial Memory</td><td>Three cups were placed in a row on the platform in front of the testing cage. Then the experimenter then showed the subject two rewards and placed them under two of the three cups in full view of the subject. Then the platform was pushed towards the subject and it was allowed to make up to two choices in succession. If, however, the subject chose the empty cup first, it was not allowed to make further choices. The response was counted as correct when the subject had chosen both baited cups in succession.</td><td>Our point 2. The "Spatial Memory: 3 Upside Down Cups and 1 Treat" test. Three identical overturned cups were placed in a row on a platform in front of the testing cage. Then, the experimenter showed a treat to the primate and placed it under one of the three cups (random choice) in such a way that the primate could fully inspect the treat. If the primate initially chose an empty cup, it would not be allowed to make further choices. An answer was considered correct when the primate chose the cup with the treat from the first attempt, thereby demonstrating the capabilities of spatial memory and concentration on the object. A successful outcome was awarded 2 points.</td><td rowspan="2">Herrmann, E.; Call, J.; Hernàndez-Lloreda, M.V.; Hare, B.; Tomasello, M. Humans Have Evolved Specialized Skills of Social Cognition: The Cultural Intelligence Hypothesis. Science 2007, 317, 1360–1366, doi:10.1126/science.1146282.

Additional materials:

https://www.researchgate.net/publication/293979708_File_S1/fulltext/56bd3f0708aed695994639f5/293979708_File_S1.pdf</td></tr>
<tr><td colspan="2">The main differences:</td><td colspan="2">1. Test Set:

We added two new tests based on the existing: “Sticker Cup” and “Memory and Associations: Sticker Cup and a Barrier”. And also, in “Spatial Memory: 3 Upside Down Cups and 1 Treat” we made it a bit harder by leaving only one treat, whereas in the original method there were two. The original battery of tests includes dozens of tasks. We have reduced the number of tasks and selected the most appropriate ones to identify the effects of interest.

2. The use of labeling:

Our battery includes the use of a sticker to label one of the cups, which adds an element of association with the visual label, whereas the original battery lacks such an element.

3. Different approaches to memory testing:

The original PCTB includes tests with simple and double cup movements, which also allows us to assess primates' ability to track object movement. However, this approach makes much more difficult for primates. On the contrary, our battery includes tests designed to test primates' ability to memorize the location of a reward under a particular cup without movement. Our battery's tests also include a training element, where</td></tr>
</table>

		primates are required to associate a sticker with a reward, and this is reinforced over the course of training. There is no explicit learning and reinforcement phase in the original battery.<br><br>4. Scoring System:<br><br>Our battery has a scoring system where successful choices are rewarded with a certain number of points (1, 2 or 4 points depending on the test and difficulty), which is not presented in the original battery. We believe that our approach will make it easier to analyze the results.		
III. Reaction to a New Object				
1.	Reaction to a New Object	The essence of this methodology is as follows: an animal is provided with an object whose key characteristic is novelty. Additionally, depending on the primate species under study, the size and durability of the object should be taken into consideration. In this particular study, a multicolored plastic cube (15x15x15 cm) was used as an object. The animal is given a specific amount of time to interact with the object, in this case, 300 seconds. The test is monitored using a video camera. The time and the type of contact with the object are recorded. Through experimental means, we identified the following types of animal-object interactions: touching, flipping, holding, dragging, shaking, picking, pushing, moving, throwing, biting, licking, inspecting, and sniffing. If contact with the object ceases, the time is recorded, and "left" is noted.<br><br>Exploratory behavior is assessed based on the following characteristics:<br>- Reaction Latency: Expressed as the duration of time from the start of the test to the first contact.	Same, but with minor changes (point «The main differences»)	Anikaev, A.; Meishvili, N.; Chalyan, V.; E.N, A. Сравнительный Анализ Исследовательской Деятельности у Самцов и Самок Зеленых Мартышек (Chlorocebus Sp.); [Comparative Analysis of Research Activities in Male and Female Green Monkeys (Chlorocebus

		- Activity: Expressed as the sum of the time intervals of the animal's contacts with the object.<br>- Activity Dynamics: Expressed as the sum of the time intervals of the animal's contacts with the object in 30-second intervals.<br>- Diversity: Expressed as the number of different types of contact with the object.<br>- Concentration: Expressed as the mean value of the time intervals of the animal's contacts with the object.		sp.)] April 18 2019. [(accessed on 2 October 2023)]. Available online: https://www.researchgate.net/publication/332548357_Sravnitelnyj_analiz_issledovatelskoj_deatelnosti_u_samcov_i_samok_zelenyh_martysek_Chlorocebus_sp
**The main differences:**		1. Types of Objects:<br>- Original Method: Uses one object (multicolored cube).<br>- Our Method: Uses two objects (a red cylinder and a blue cone), one of which is familiar and the other is new.<br><br>2. Procedure:<br>- Original Method: The animal interacts with the new object immediately.<br>- Our Method: Includes preliminary training with Object A (three times for memorization) and then testing with Objects A and B after manipulations (saline injection and blood sampling).<br><br>3. Presentation Intervals:<br>- Original Method: No repeated presentations.<br>- Our Method: Object A is presented 10 days after the first time, then the next day, and several days after the manipulations.<br><br>4. Types of Contacts:<br>- Original Method: Assesses various types of contacts (touching, flipping, holding, etc.).<br>- Our Method: Records only the total time and the type of contact.<br><br>5. Additional Parameters:		

		- Original Method: Includes assessment of activity dynamics and contact diversity.<br>- Our Method: Assesses only the main parameters (reaction latency, activity, and concentration).<br><br>Conclusion:<br>Our method includes preliminary training and repeated presentations, which allows the animals to become familiar with the object before the main test and to evaluate the dynamics of their exploratory activity at multiple stages. This allows for comparison of results before and after manipulations.		
IV. Research Activity. The "Box" Test				
1.	The "Box" Test	The primate had to retrieve a bait from the box using a tool.	We proposed our own simplified version for long-tailed macaques. The "Box" test is a method designed to test the ability of primates to solve intellectual and memory tasks.	https://link.springer.com/article/10.1007/s10071-004-0239-6
V. Tiredness and Performance Capacity. The "Tiredness" Test Battery				
1.	The "Tiredness" Test	Primates were asked to take video tasks. The effect of social isolation on primates was studied. One of the effects of isolation was to performance disruption.	We estimated the parameter Tiredness by assessing the changes in performance disruption dynamics in the cascade of cognitive tests. Our approach is convenient because it allows us to additionally estimate the fatigue parameter without resorting to the use of additional tests, which facilitates the work of the experimenters, as well as does not overload the primate program and does not involve any medical procedures (motor endurance tests and physiological measurements are not used), including minimising subjectivity (behavioural observations).	https://pubmed.ncbi.nlm.nih.gov/1860309/
